# Genetic divergence and phylogeographic history of two closely related species (*Leucomeris decora* and *Nouelia insignis*) across the 'Tanaka Line' in Southwest China

**DOI:** 10.1186/s12862-015-0374-5

**Published:** 2015-07-08

**Authors:** Yu-Juan Zhao, Xun Gong

**Affiliations:** Key Laboratory for Plant Diversity and Biogeography of East Asia, Kunming Institute of Botany, Chinese Academy of Sciences, Kunming, 650204 China

**Keywords:** *Leucomeris decora*, *Nouelia insignis*, Plastid DNA, Nuclear DNA, Genetic divergence, Migration, Tanaka line

## Abstract

**Background:**

*Leucomeris decora* and *Nouelia insignis* (Asteraceae) are narrowly and allopatrically distributed species, separated by the important biogeographic boundary Tanaka Line in Southwest China. Previous morphological, cytogenetic and molecular studies suggested that *L. decora* is sister to *N. insignis*. However, it is less clear how the two species diverged, whether in full isolation or occurring gene flow across the Tanaka Line. Here, we performed a molecular study at the population level to characterize genetic differentiation and decipher phylogeographic history in two closely related species based on variation examined in plastid and nuclear DNAs using a coalescent-based approach.

**Results:**

These morphologically distinct species share plastid DNA (cpDNA) haplotypes. In contrast, Bayesian analysis of nuclear DNA (nDNA) uncovered two distinct clusters corresponding to *L. decora* and *N. insignis*. Based on the IMa analysis, no strong indication of migration was detected based on both cpDNA and nDNA sequences. The molecular data pointed to a major west-east split in nuclear DNA between the two species corresponding with the Tanaka Line. The coalescent time estimate for all cpDNA haplotypes dated to the Mid-Late Pleistocene. The estimated demographic parameters showed that the population size of *L. decora* was similar to that of *N. insignis* and both experienced limited demographic fluctuations recently.

**Conclusions:**

The study revealed comprehensive species divergence and phylogeographic histories of *N. insignis* and *L. decora* divided by the Tanaka Line. The phylogeographic pattern inferred from cpDNA reflected ancestrally shared polymorphisms without post-divergence gene flow between species. The marked genealogical lineage divergence in nDNA provided some indication of Tanaka Line for its role as a barrier to plant dispersal, and lent support to its importance in promoting strong population structure and allopatric divergence.

**Electronic supplementary material:**

The online version of this article (doi:10.1186/s12862-015-0374-5) contains supplementary material, which is available to authorized users.

## Background

The Quaternary climate fluctuation largely influenced the distribution ranges of plants, leading to range expansion, contraction and/or population extinction [1]. Range expansion can result in contacts between previously geographically separated species and lead to profound consequences for lineage or species evolution, as they may cause introgression between species and haplotypes sharing across species. Range contraction of widespread species may have left isolated populations in marginal areas, providing chances for peripheral speciation of regional endemics [2–5]. It is assumed that gene transfer events may have occurred between two species in previously sympatry or parapatry populations, when species-specific haplotypes are found in another with allopatric distribution [4, 6]. In addition, sharing of haplotypes across species can not only be caused by introgression but also by incomplete lineage sorting. The boundaries between species can be blurred due to the existence of introgression and/or retention of ancestral polymorphism [7].

Generally, in case of incomplete lineage sorting, we would expect the shared cytoplasmic-nuclear haplotypes to be randomly distributed in a mosaic pattern over the species geographic range [8, 9]. In contrast, if haplotypes sharing between both species arises as a function of introgression, we would expect co-occurring populations of the two species are more similar than randomly chosen population pairs [5, 10, 11]. Recent coalescent methods like IM model (Isolation with Migration) based on sequencing loci from different inherited systems can also help to address these questions by estimating the extent of genetic divergence and revealing demographic dynamics by distributional changes, which are applied to within species or between recently divergent taxa [12, 13].

*Nouelia insignis* Franch. and *Leucomeria decora* Kurz, both belonging to the Mutisieae (Asteraceae) [14], are characterized by woody growth form and rare in China. *N. insignis* is a monotypic genus endemic to southwest China and *Leucomeris* contain two extant species, one of which occurred in Yunnan China. *N. insignis* and *L. decora* are not sharply divergent morphologically but differ most prominently in flora traits. *L. decora* has capitula in a dense terminal cyme and tubular florets, whereas *N. insignis* is characterized with numerous, solitary, terminal and radiate capitula, and fertile florets with marginal uniseriate, bilabiate florets, and central tubular florets [15]. Despite the little distinctively morphological differentiation, they are closely related phylogenetically: *Leucomeris* and *Nouelia* were considered to be independent and treated as *Nouelia* group based on morphological characters [14, 16]; and then molecular studies based on chloroplast DNA variation in Panero and Funk showed they clustered together within a clade that also contains the South American genera *Hyalis* and *Ianthopappus* [17]; later Funk et al. considered them a ‘*Leucomeris*’ clade [18]. Particularly, a previous study inferred a sister relationship between these two species based on molecular phylogeny (cpDNA and nDNA) of eastern Asian Mutisieae [19]. Moreover, the two species have the same chromosome numbers (2n = 54), and this character distinguishes them from the other genera in the tribe Mutisieae [20]. Together, all these suggest that *L. decora* and *N. insignis* are tightly related, differing from other Asian or American species of Asteraceae.

*L. decora* and *N. insignis* are allopatric over their natural ranges. *L. decora* has a large distribution range, extending from south Asian countries like Myanmar, Thailand and Vietnam to China, whereas *N. insignis* has a more restricted distribution in Yunnan and Sichuan in China [21]. They both can grow in valleys with dry and hot environmental condition; however, in contrast, *L. decora* also prefers the edge of the forest and isolated mountaintops [22, 23]. In particular, the distribution ranges are found to coincide with known the biogeographic boundary, i.e. Tanaka Line, which separates two environmentally divergent subkingdoms, that is the ‘Sino-Himalayan Forest’ to the west and the ‘Sino-Japanese Forest’ to the east [24–26]. Although it is not an apparent physical barrier like mountain ranges, river systems or sea straits, deep divergences between the boundary have been reported (such as [27–30]). Taking the reason into account, one recent phylogeographic study supported that the dramatic climate changes during the (Late) Pleistocene, when the presently differing monsoon regimes on either side of the Tanaka Line established, may be a reasonable explanation for the divergence [31]. However, for studies with sufficient sampling from areas proximal to and/or along the Tanaka Line are still lacking, assessing the feature as a genetic boundary remains largely hypothetical.

Based on the distribution range and closely related relationship, these two species may provide a model to study the present genetic variation and assess whether there is long-term population isolation without migration across the Tanaka Line. Although, previous study using molecular sequences hypothesized the recent origins and phenotypic evolution via adaptation to dry and cool habitats during the Quaternary glaciation; however, only a small number of populations of *L. decora* and *N. insignis* and only one individual per population were sampled in the above molecular investigation. Thus, more comprehensive analyses based on a sufficient population sampling of both species are needed to address the population genetic differentiation and to investigate historical demography of the two species. In the present study, we compared sequence variation at both cpDNA fragment and the low-copy nuclear gene, across a large number of individuals and populations covering the natural ranges of *L. decora* and *N. insignis* in China. Specifically, we aim to determine the amount of sequence divergence and possible gene flow after divergence between *L. decora* and *N. insignis*, and to disentangle the extant genealogical lineage patterns possibly associated with the Tanaka Line, and attempt to explore their demographic history. Together, these analyses should add our understanding of allopatric species divergence across Tanaka Line and the possible explanation for the Tanaka Line as a biogeographic boundary in southwest China.

## Methods

### Population sampling

Fresh leaves of *L. decora* and *N. insignis* were collected in the field and dried directly with silica gel. We sampled 11 natural populations of *L. decora* and 16 of *N. insignis*, respectively, covering the distributional range of the two species in China. In total, 251 individuals from 27 populations were surveyed for chloroplast and nuclear DNA variations. Voucher specimens were collected and deposited in the Herbarium of Kunming Institute of Botany, Chinese Academy of Sciences (KUN). Detailed information on the geographical distribution of the two species and the localities of the populations sampled in this study is shown in Additional file 1: Table S1 and Additional file 2: Table S2.

### DNA extraction, PCR amplification, DNA sequencing and cloning

Genomic DNA was extracted from silica-dried leaves using the CTAB method [32] with some modifications. One chloroplast DNA intergenic spacer *rpl32-trnL*^*(UAG)*^ [33] was amplified and sequenced. We followed our previous study in sequencing chloroplast DNA [34]. In addition, we selected one single or low-copy nuclear locus to estimate the genetic diversity and geographic structure of the two species. The granule-bound starch synthase gene (*GBSSI* or *WAXY*) has been reported to be single–copy in diploid grasses [35, 36] and several dicots [37, 38]. Recently, it has been broadly used in molecular study [39–41].

For the *GBSSI* gene, it was amplified and sequenced using the pair of primer *GBSSI*_*2*_*F* (5’-ACA TTG CYT ACC AAG GNA GA-3’) and *GBSSI*_*2*_*R* (5’-AAC TGA ATG AGA CCA CAM GG-3’) in the preliminary phase of the study, designed from the complete coding sequence of *Ipomoea batatas* (Conolvulaceae, AB071976) [42] and its homologous sequences using the program Primer Premier 5.0. The obtained sequences were blasted for sequence homology to ensure that they were *GBSSI* genes encoding granule-bound starch synthase. Then one pair of primer *GBSSIF*: (5’-TTG ATT TCA TTG TTG ATG GGT-3’) and *GBSSIR* (5’-GGG CCA GTT GCA CAT TGA ATT TAG C-3’) was designed according to the previously obtained sequences for amplification and sequencing, which was conducted in a similar way to that described by previous study [34].

As for the nuclear gene region, some individuals with only one heterozygous site were directly split into two alleles through haplotype subtraction [43], while products from individuals with more than 2 heterozygous sites (according to the first sequencing round) were re-sequenced to eliminate sequencing errors. Products from any other individuals that could not be directly sequenced or had more than 2 heterozygous sites according to the second sequencing round were cloned.

### Analyses of variation for plastid and nuclear DNAs

Multiple alignments of the sequences were obtained using Clustal X 1.81 [44] and then improved manually by BioEdit [45]. Molecular diversity indices, including the number of haplotypes, haplotype diversity (Hd), nucleotide diversity (π) [46] were estimated for the species and each population using DnaSP program version 3.95 [47]. The total gene diversity (H_T_) and the average gene diversity within populations (H_S_) were calculated for *L. decora* and *N. insignis* for cpDNA markers. Two measures of genetic differentiation within two species, G_ST_ (coefficient depending only on the frequencies of the haplotypes) and N_ST_ (coeffient of genetic variation influenced by both haplotype frequencies and genetic distances between haplotypes) were estimated following Pons and Petit [48] as implemented in the program PERMUT. Genetic differentiation between the two species was assessed in two ways. First, hierarchical partitioning of diversity among species, populations and individuals was estimated on the basis of AMOVA in Arlequin 3.1 with significance tested using 1000 permutations [49]. Secondly, a Bayesian clustering analysis using STRUCTURE ver. 2.3 was conducted to assess population structure at nuclear locus for all samples. An admixed model was used, and the correlated allele frequencies among clusters were assumed. To estimate the number of clusters (K), values of K from 1 to 10 was set using 20 independent runs per K to test the stability of the results. The optimal K was estimated according to the model value (ΔK) following the procedure described by [50].

### Phylogenetic analyses and dating TMRCA of cpDNA lineages

In order to infer the interspecific relationships between the two species, cpDNA and nDNA haplotypes were reconstructed using statistical parsimony as implemented in TCS with the connection limit set to 95 % and gaps treated as fifth character [51]. For this analysis, substitution and indels were assumed to evolve with equal possibility although they may exhibit different mutation rates. And indels longer than 1 bp were coded as substitutions and mononucleotide repeats were removed due to their high degree of homoplasy [52]. The time of the most recent common ancestor was estimated using Bayesian inference on the cpDNA sequence *rpl32-trnL* haplotypes as implemented in BEAST v.1.5.4 [53]. We used a HKY substitution model, selected by jModelTest [54], a strict molecular clock and a Yule process as tree prior. We assumed minimum and maximum values of a range of average mutation rates reported for synonymous sites of plant chloroplast genes [i.e., 1.2 and 1.7 × 10^-9^ substitutions per site per year (s/s/y)] for the BEAST analysis [55]. Three separate MCMC runs were performed, each of 1 × 10^7^ generations, with sampling every 1000th generation, following a burn-in of the initial 10 % cycles. Convergence of the parameters sampled was checked in TRACER v.1.5 and combined with Log-Combiner v.1.5.4 [56].

### Neutrality tests and mismatch distribution analyses

In order to test whether historical expansion events have ever occurred in *L. decora* or *N. insignis*, two neutrality tests were performed: (1) Tajima’s *D* considering the frequency of mutation (segregating sites) [57], and (2) Fu’s *Fs* based on the haplotype distribution [58]. The demographic history of a population could be inferred by comparing such neutrality tests, given that a range expansion is suggested when Tajima’s *D* and Fu’s *Fs* are significantly negative. The statistical significance of these estimates was calculated with 1000 permutations. In addition, the molecular distances among sequences were estimated through pairwise differences. The distribution of these differences (mismatch distribution) was used to test two population expansion models, in which cases unimodal distributions are expected [59]. We tested the null hypotheses of a spatial expansion and a pure demographic expansion through the sum of squares deviation (SSD) between the observed and the expected distributions and through the Harpending’s raggedness index, which were used to test the goodness-fit of the observation mismatch distribution to the expectation of expansion models. All the above analyses were implemented in Arlequin.3.1 [49].

### Demographic parameters of the IM model

Divergence time between the two species and effective population sizes as well as interspecific gene flow by calculating relative migration rates between species were estimated using an isolation-with-migration (IM) model in IMa version 12/19/2009 [60]. This model uses Markov chain Monte Carlo (MCMC) to estimate the posterior probability densities of estimated parameters. It involves several simplifying assumptions such as neutrality and no recombination within loci, lack of genetic contribution from unsampled populations, and random mating in ancestral and descendent populations. [60, 61]. It is difficult to satisfy all of these assumptions in an empirical study. We used the program IMgc [62] to analyze recombination within the nuclear locus, and the only maximally informative and recombination-free datasets identified were included in the final analysis. IMa was run for the entire dataset, including all sampled nuclear sequences without recombination and chloroplast sequences. We performed preliminary simulations to assess convergence of the MCMC chains on the data stationary distribution and only estimates whose posterior distribution dropped to zero within the prior intervals investigated were trusted. For each analysis, burn-in was set to 10 million steps, and a total of 50 million steps were conducted. To verify convergence on the same parameter values, we ran the analysis three times with different random seeds. Demographic parameters were scaled to the generation time and neutral mutation rate. We used a generation time of five years in both *L. decora* and *N. insignis* as observed in the field and under cultivation. In the absence of a well-calibrated estimate in *L. decora* and *N. insignis*, we applied a generic average mutation rate of 1.45 × 10^-9^ substitution per site per year (1.2 and 1.7 × 10^-9^) for cpDNA and 6.1 × 10^-9^ (5.1 and 7.1 × 10^-9^) for nDNA [55]; and the geometric average mutation rate of the two marker sets was used to scale the outputs to demographic units.

## Results

### Chloroplast haplotype lineages and dating TMRCA

**Fig. 1:**
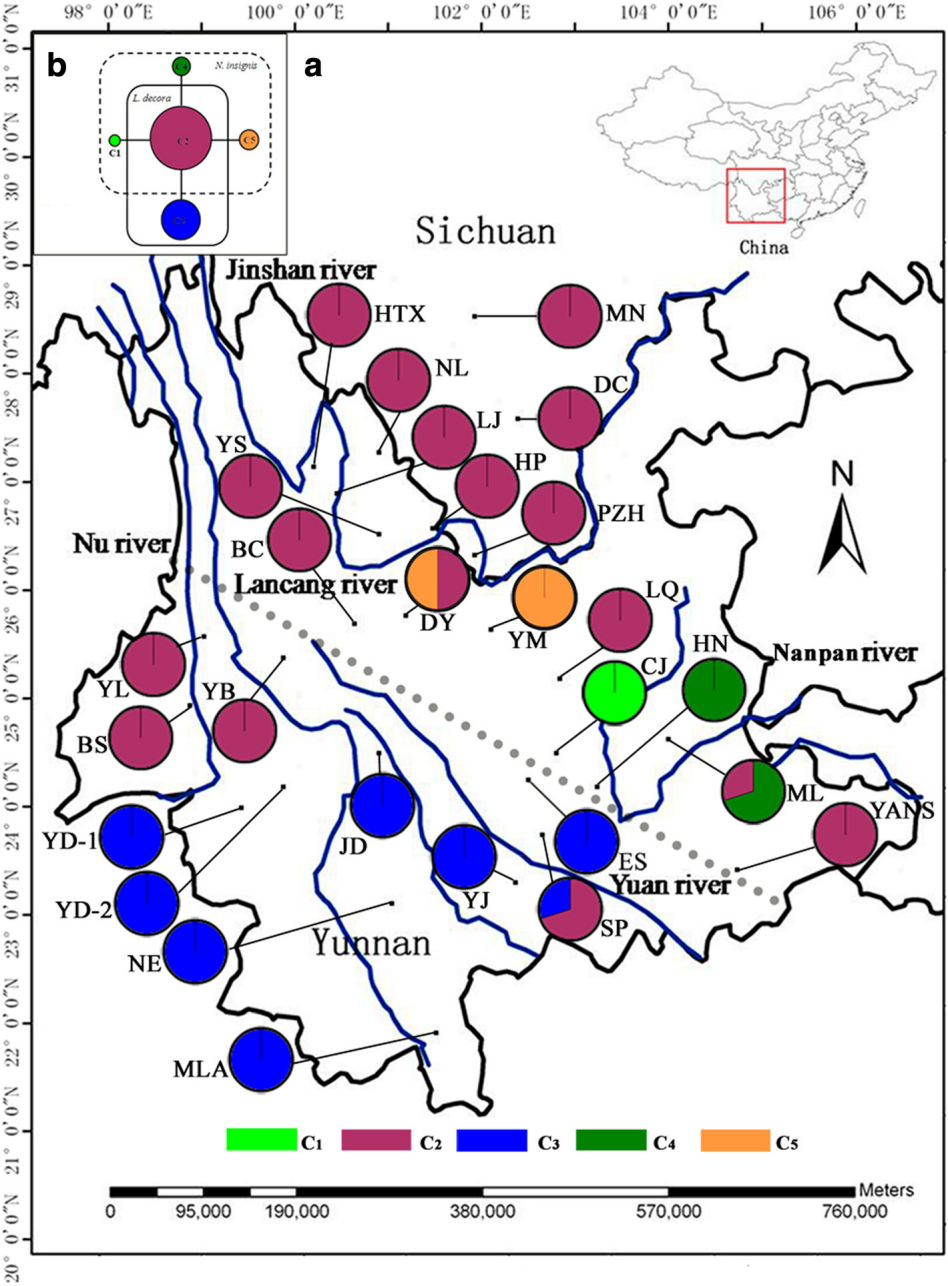
Geographic distribution and network of cpDNA haplotypes. **a**. Distribution of 5 chloroplast DNA (cpDNA) haplotypes detected within and among 27 populations of *Leucomeris decora* and *Nouelia insignis*. Full names of the abbreviations for the populations are shown in Additional file 1: Table S1 and Additional file 2: Table S2. **b**. Statistical parsimony network of genealogical relationships between five cpDNA haplotypes. Letters in/around circles represent haplotypes at each locus. The size of the circles corresponds to the frequency of each haplotype and each solid line represents one mutational step

One indel and three substitutions were detected from the cpDNA region *rpl32- trnL*^*(UAG)*^ (894-900 bp excluding the mononucleotide repeats) of *L. decora* and *N. insignis* samples, and were recoved 5 chloroplast haplotypes (C1-C5). All haplotype sequences were deposited in GenBank databases under the accession numbers JF915764–JF915768. The most common haplotype C2 (frequency 62.1 %) was shared by the two species, and 37 %, 78 % of the investigated individuals carried this haplotype in *L. decora* and *N. insignis*, respectively. C3 (24.7 %) only occurred in *L. decora* and the other haplotypes (C1, C4, C5) were only found in *N. insignis* with low frequencies ranging from 1.95 % to 5.98 % (Additional file 1:Table S1 and Additional file 2: Table S2). The distribution and network of cpDNA haplotypes C1–C5 are shown in Fig. 1. The network of chloroplast haplotypes indicated that C2 was possibly ancestor to the others and away from the remaining haplotypes by a single mutation (Fig. 1b). And it was fixed in most of *N. insignis* populations located in the Jinsha drainage and northwestern populations of *L. decora* (Fig. 1). All the remaining populations of *L. decora* in centre/southwest Yunnan were fixed for C3 except one population SP. C1 and C4 were restricted to the Nanpan drainage (CJ, HN and ML) and C5 was only found in population DY and YM. The total cpDNA diversity was slightly higher in *L. decora* (H_T_ = 0.487) than in *N. insignis* (H_T_ = 0.422). Between-population differentiation levels estimated by both G_ST_ and AMOVA are relatively low within *N. insignis* (G_ST_ = 0.849, F_ST_ = 0.896) than in *L. decora* (G_ST_ = 0.862, F_ST_ = 0.907) (Tables 1, 2). The Bayesian TMRCA analysis indicated that all sampled cpDNA haplotypes of *L. decora* and *N. insignis* coalesce at about 0.54 Ma (95 % confidence interval: 0.01–1.32 Ma) or 0.38 Ma (95 % confidence interval: 0.01–0.93 Ma), assuming minimum and maximum (average) rates of synonymous substitution in cpDNA, respectively. This suggests that the divergence between *L. decora* and *N. insignis* falls into the mid-late Pleistocene.

**Table 1:** Different hierachical types of analysis of molecular variance (AMOVA) of Leucomeris decora and Nouelia insignis

Markers	Source of variation	d.f.	Sum of squares	Variance	% Total	Fixation index
cpDNA	Between species	1	24.9	0.18	35.5	F_CT_ = 0.355**
	Among populations	25	70.9	0.30	58.7	
	Within populations	224	6.7	0.03	5.8	F_ST_ = 0.942**
*L. decora*	Among populations	10	21.1	0.23	90.7	F_ST_ = 0.907**
	Within populations	88	2.1	0.02	9.3	
*N. insignis*	Among populations	15	49.9	0.35	89.6	F_ST_ = 0.896**
	Within populations	136	5.5	0.04	10.4	
nDNA	Between species	1	239.0	0.98	68.4	F_CT_ = 0.684**
	Among populations	25	78.3	0.15	10.6	
	Within populations	475	143.0	0.30	21.0	F_ST_ = 0.79**
*L. decora*	Among populations	10	33.9	0.16	26.3	F_ST_ = 0.263**
	Within populations	187	86.0	0.46	73.7	
*N. insignis*	Among populations	15	44.5	0.15	42.4	F_ST_ = 0.424**
	Within populations	288	57	0.20	57.6	

d.f., degree of random; **P < 0.001

### Nuclear DNA variation and haplotype lineages

**Fig. 2:**
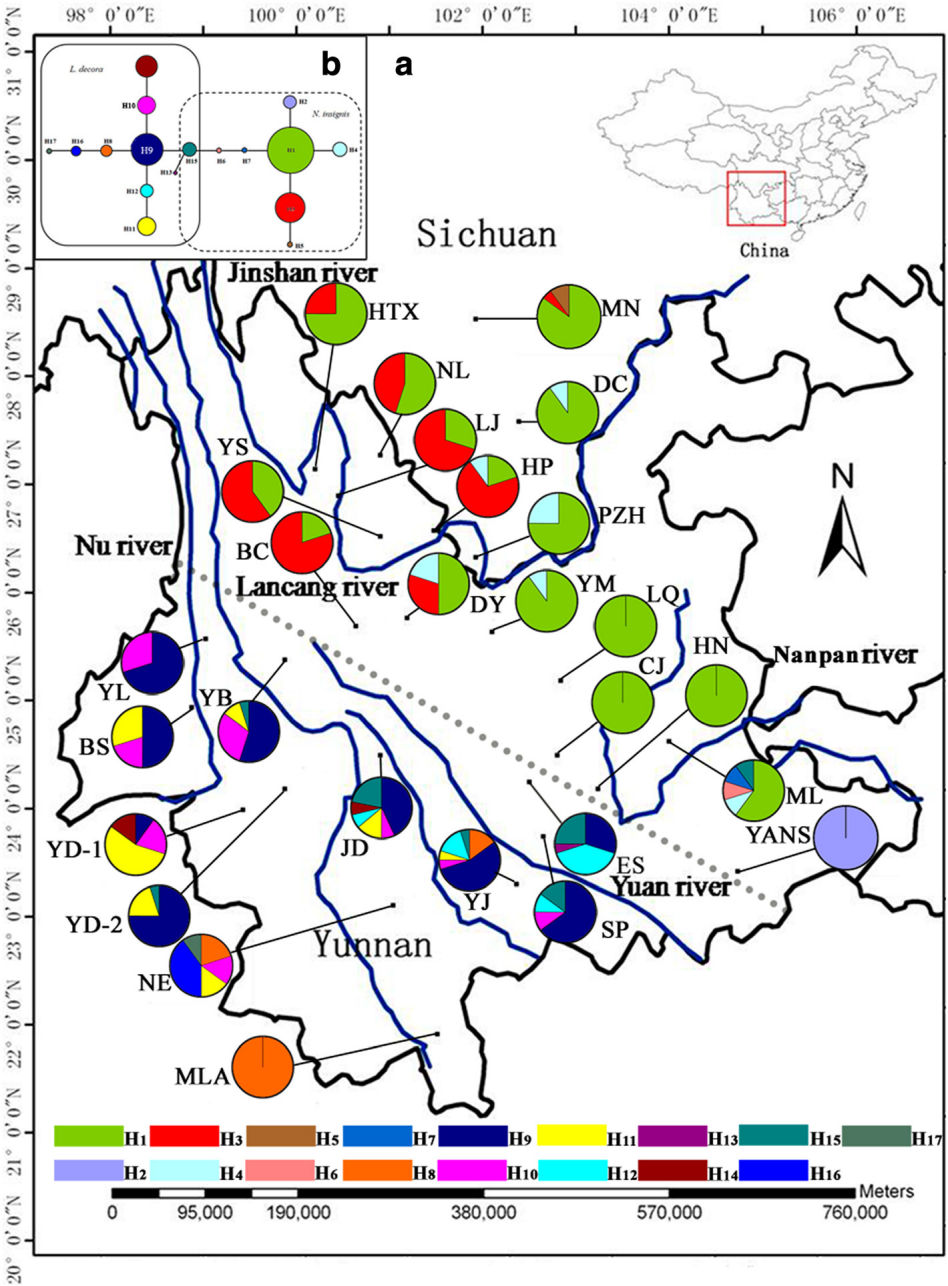
Geographic distribution and network of nDNA haplotypes. **a**. Distribution of 17 nuclear DNA (nDNA) haplotypes detected within and among 27 populations of *Leucomeris decora* and *Nouelia insignis*. Full names of the abbreviations for the populations are shown in Additional file 1: Table S1 and Additional file 2: Table S2. **b**. Statistical parsimony network of genealogical relationships between 17 nDNA haplotypes. Letters in/around circles represent haplotypes at each locus. The size of the circles corresponds to the frequency of each haplotype and each solid line represents one mutational step

Among the 251 individuals of *L. decora* and *N. insignis*, 214 and 37 were homozygotes and heterozygotes, respectively. A total of 17 genotypes (alleles) were recognized based on 9 substitutions in the sequence alignment (688 bp). All haplotype sequences were deposited in GenBank databases under the accession numbers JF915747–JF915763. The distribution and network of nDNA haplotypes H1-H17 are shown in Fig. 2. In the network, several closed loops, perhaps resulted from recombination, were resolved using the rules given by Templeton and Sing [63] and Posada and Crandall [64]. Seven haplotypes (H1–H7) were only found in *N. insignis* in which H1 was the most common haplotypes with frequency larger than 60 %. The rest of the haplotypes (H8–H17) all occurred in *L. decora* except for H15, which was also observed in one individual in the population ML of *N. insignis*. The dominant haplotype H9 had widespread distribution in 9 populations of *L. decora* and accounted for 44.4 % of the total haplotypes identified in the species (Fig. 2b). In addition, the network without recombination within this locus was also constructed and a similar genealogy was found, with approximately two clusters with haplotypes being species-specific except Hap5 (Additional file 3: Fig. S1).

### Genetic differentiation and population structure

**Fig. 3:**
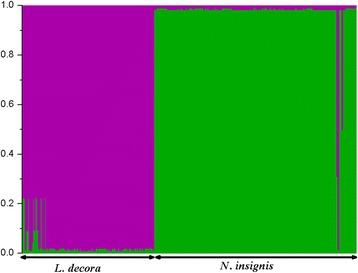
Clustering analysis of nDNA for *Leucomeris decora* and *Nouelia insignis* populations using STRUCTURE. Each bar of the plot represents one individual, with estimated likelihood assignment on the y-axis, when K = 2 cluster is assumed

The nucleotide diversity (π) and haplotype diversity (Hd) of each population from both markers in the two species was generally low (Additional file 1: Tables S1 and Additional file 2: Table S2). At the species scale, *L. decora* had relatively higher nucleotide diversity and haplotype diversity than those of *N. insignis* (Table 2). The AMOVA analyses of cpDNA and nDNA datasets showed significant genetic differences between species as well as among populations (Table 1). For example, the difference between the two species explained 68.4 % of the total nDNA variation and 35.5 % of cpDNA. Based on the cpDNA data, in *L. decora* 90.7 % was attributed to differences among populations and 9.3 % was attributed to differences among individuals within the population; and in *N. insignis*, 92.5 % was attributed to differences among populations and 7.5 % was attributed to differences among individuals within the population. In the Bayesian clustering implemented by STRUCTURE, the most likely number of clusters was two when the Δ*K* statistic of Evanno et al. [50] was applied (Additional file 4: Fig. S2). When *K* = 2, all individuals of *L. decora* and *N. insignis* formed two separate clusters, except for admixed individuals present in several populations, especially one individual (population 26) of *N. insignis* (Fig. 3). These patterns of individual assignments were consistent with the result of genealogy.

**Table 2:** The total *rpl32-trnL* and *GBSSI* variation, genetic diversity and differentiation parameters in all populations of *Leucomeris decora* and *Nouelia insignis*. π: nucleotide diversity and Hd: haplotype diversity

	*rpl32-trnL*						*GBSSI*	
Species	π × 10^-3^	Hd	H_T_	H_S_	G_ST_	N_ST_	π × 10^-3^	Hd
*L. decora*	0.53	0.473	0.487 (0.0935)	0.042 (0.0424)	0.913 (0.0850)	0.913 (0.0850)	1.67	0.751
N. insignis*	0.38	0.32	0.422 (0.1376)	0.064 (0.0438)	0.849 (0.1085)	0.862 (0.1009)	0.97	0.550

Standard error is shown in parentheses. *indicates the loci which exhibited significantly larger N_ST_ than G_ST_

### Neutrality tests and mismatch distribution analyses

Neither the Tajima’s *D* nor Fu’s *Fs* neutrality tests yielded significantly negative results for all *L. decora* populations or *N. insignis* populations (Table 3). Similarly, the mismatch distribution was multimodal for *L. decora* populations with uniformly significant SSD and H_Rag_ values. However, the unimodal distribution pattern with non-significant SSD and H_Rag_ values was presented by all *N. insignis* populations under the demographic expansion model (Fig. 5). All these observations indicate the absence of expansion events.

**Table 3:** Summary of mismatch distribution parameters and neutrality tests for all populations of *Leucomeris decora* and *Nouelia insignis*, respectively

	*rpl*32-*trnL*					
Species	Tajima’s D	Fu’s Fs	SSD^a^	H_Rag_^a^	SSD^b^	H_Rag_^b^
*L. decora*	1.659	2.187	0.017*	0.227*	0.017*	0.227*
*N. insignis*	-0.083	0.793	0.005*	0.194	0.012	0.194

SSD, the sum of square deviation; H_Rag_, the raggedness index; *P < 0.05

^a^The indices under the spatial expansion model; ^b^The indices under the sudden demographic expansion model

Estimates were obtained under models of spatial expansion or pure demographic expansion using Arlequin

### Demographic parameters of the IM model

**Fig. 4:**
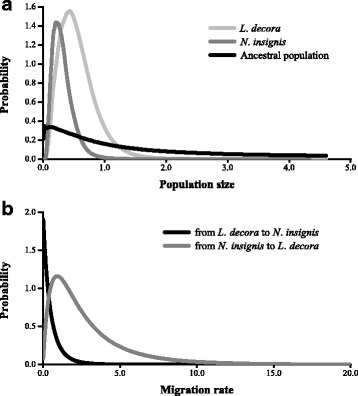
Marginal distribution of posterior probabilities for demographic parameters estimated by IM model. The IM analysis was performed for combined cpDNA and nDNA sequences. **a**. Population sizes of *Leucomeris decora*, *Nouelia insignis* and their ancestral population; **a**. migration rate between *Leucomeris decora* and *Nouelia insignis*

For three independent runs of IMa, consistent unambiguous marginal posterior probability distributions of most demographic parameters were obtained for the two species (Fig. 4). However, the marginal posterior probability distributions of the parameter *t* did not drop to zero when sufficient high values were reached. This estimated result was likely due to small data set that may not be enough to achieve convergence [65]. The maximum-likelihood estimates (MLEs) and 90 % highest posterior densities (HPDs) were shown in Additional file 5: Table S3. Effective population sizes of *L. decora* and for *N. insignis* were not significantly different and larger than that of the ancestral species, indicating that both *L. decora* and *N. insignis* have undergone marked population expansion. The estimates of migration between *L. decora* and *N. insignis* were low and asymmetric. The gene flow from *N. insignis* to *L. decora* was larger than zero: m_2_ = 0.228 (90 % HPD interval: 0.008–1.563), while there was almost no evidence for gene flow in the opposite direction: *m*_1_ = 0.01 (90 % HPD interval: 0.01–1.41). The population migration rate was 2*N*_2_*m*_2_ = 0.049 from *N. insignis* to *L. decora* and 2*N*_*1*_*m*_*1*_ = 0 from *L. decora* to *N. insignis*, respectively.

## Discussion

### Haplotype lineage and cytoplasmic-nuclear incongruence

Our results showed that the nDNA haplotypes (Fig. 2) were more species specific in comparison with the cpDNA sequences that we analysed (Fig. 1). Only H15 was shared by both species, which was frequent in *L. decora*, but found in only one individual of *N. insignis*. The network without recombination within the nuclear locus revealed a similar pattern, in which *L. decora* and *N. insignis* comprised different haplotypes and no haplotypes were shared except Hap5 (Additional file 3: Fig. S1). However, the cpDNA analyses provided an unexpected result with that one dominant haplotype (C2) detected from cpDNA and shared by *L. decora* and *N. insignis* (Fig. 1). There are three main causes of species sharing haplotypes: convergence, the persistence of ancestral polymorphism [66], or cryptic hybridization/introgression [67, 68].

Convergence appears to be the least likely explanation in this case as identical mutations are rare events, and the common haplotype with high frequency of more than 60 % of the individuals was present in the two species. Gene exchange caused by ancient and/or current hybridization between *L. decora* and *N. insignis*, resulting in the cpDNA capture, is possible. Sharing of haplotypes between two potentially hybridizing species only in areas where they are sympatric would lend support to the local hybridization hypothesis. However, in the process of thoroughly sampled populations of the two species, no overlapping distribution areas and no signs of intermediate morphology for species-specific taxonomic traits were found either at present; current hybridization between these two species seems unlikely to have played a major important role in causing the sharing of cpDNA haplotypes across them [69]. An alternative scenario is that hybridization and chloroplast capture may have occurred in the past when these two species came into contact with each other. Nevertheless, in the thoroughly sampled populations, chloroplast types were mostly distinct in the two species where they occurred allopatrically and the shared haplotype C2 was not restricted to some areas but broadly distributed in the populations of the two species. In fact, in 27 populations, we found only one haplotype was shared, and other haplotypes were in a relatively derived position in the network. Thus, as mostly interior haplotypes, if at all, are shared among species and shared chloroplast types also occur between species without overlapping distribution areas, persisting ancient polymorphisms seem to be the most likely explanation for the extant chloroplast patterns in *N. insignis* and *L. decora*. The finding of old cpDNA haplotypes shared may also indicate a recent divergence between these two closely related taxa and underwent an allopatric phase of divergence.

### High levels of genetic differentiation and little gene flow between *L. decora* and *N. insignis*

Strong genetic differentiation across the Tanaka Line has been reported previously, however, it should be noted that such molecular data were derived from one species or species complexes; comparative analyses of closely related species distributed along the Tanaka Line remain still tenuous. Based on our data set, despite the sharing of one high frequent cpDNA haplotype, we detected high levels of genetic differentiation between *N. insignis* and *L. decora* populations (Table 1). Firstly, some cpDNA and *GBSSI* haplotypes that were frequent in *N. insignis* (e.g., C5, H1 and H3) were not found in *L. decora* and vice versa. Secondly, AMOVA produce that 35.5 % and 68.4 % of the total cpDNA and nDNA genetic variation could be attributed to differences between the two species, respectively. These observed genetic differentiation coefficients were relatively high by comparison with those of closely related species (see [70–72]). Moreover, in the STRUCTURE analyses, the absence of genetic admixture and robust population clustering in the two species also indicate high genetic differentiation (Fig. 3).

The likelihood ration test from coalescent-based analyses (IM model) indicated no gene flow from *L. decora* to *N. insignis* but very little gene flow from *N. insignis* to *L. decora* for most of the pairwise comparisons. Introgression events between closely related species are widespread in plants. Introgression within genera was far more common than that between genera [73]. Recent phylogenetic studies have also discovered intergenetic gene transfer, which played an important role in lineage evolution [74–76]. Gene flow between the two species would have seem likely because the distance between some of our samples, like HN and ES, is only about 15 km. The seeds with the light pappus may disperse by wind across the Tanaka Line. Moreover, even if the two species are currently allopatric, the possibility that the two species may have sympatric distributions and one of them went extinct in the sympatric areas is not completely excluded. Accordingly, they may not have diverged from each other in geographic isolation or subsequently always been allopatrically distributed. However, although there was unidirectional gene flow observed in the two species that are currently allopatric, the population migration rate (2*N*_2_*m*_2_) from *N. insignis* to *L. decora* was very low (0.049 < 1.0), suggesting that historical dispersal between populations seemed rare (Fig. 4, Additional file 5: Table S3). The recovered signals of close to zero migration, therefore, also indicated that the high levels of cpDNA haplotype sharing observed are most likely due to incomplete lineage sorting and widespread sharing of ancestral polymorphism, and the two species have been isolated genetically and well-differentiated since their divergence [77].

Generally, physical barriers like mountain ranges and river systems may act as physical barrier to past or contemporary dispersal. However, deep interspecific genetic divergences along the Tanaka Line without apparent physical barriers have not been reported. Most frequently, biogeographic boundaries are explained to be associated with some combination of historical and ecological processes [78], causing lineage divergence and local endemism (such as [79]). It has been proposed that monsoon characterized the two sides of the Tanaka Line (i.e., the west affected by monsoons from both the Indian and Pacific oceans and the east mainly affected by Pacific oceans) may result in the separation of species population. Presently, *N. insignis* and *L. decora* populations occupy different habitats on the two sides of the Tanaka Line. *N. insignis* prefers drier and hotter conditions such as valleys in the east, whereas *L. decora* is widely distributed in the west. It is feasible that the establishment of this biogeographic boundary corresponds with formation of the monsoon systems between the two sides, which promoted initial divergence between these two species (see [31] and discussion below).

### Phylogeographic history of *L. decora* and *N. insignis*

**Fig. 5:**
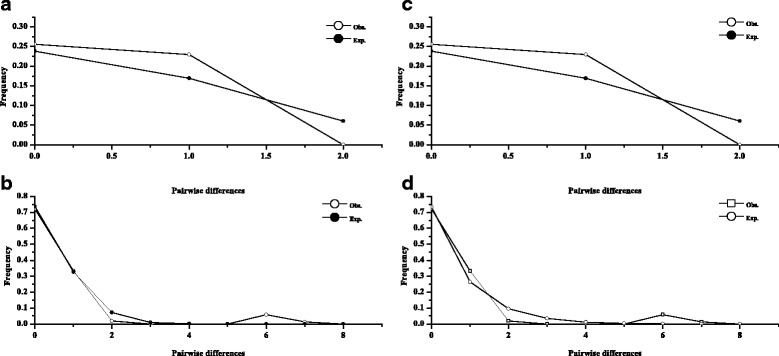
Mismatch distribution for cpDNA sequence data in *Leucomeris decora* **(a, b)** and *Nouelia insignis* **(c, d)**, under the spatial expansion model **(a, c)** and demographic expansion mode **(b, d)**, respectively. The line with hollow dot shows observed values, while the line with solid dot represents expected values under the model of spatial/demographic expansion

The coalescent parameters of the divergence time with the MLEs and HPD weakly defined peaks and with undefined upper boundaries, cannot be used for scaling the precise divergence time between *L. decora* and *N. insignis*, perhaps due to the small data set that did not result in their convergence [80]. However, our crude estimated time frame (c. 0.38–0.54 Ma for cpDNA) for the TMRCA of these two species could date to Mid-Late Pleistocene, when Southwest China has experienced climatic changes such as establishment of differing monsoon regimes on either side of the Tanaka Line [27, 31, 81]. Although the main factors initiating the west-east split between the two species remain unclear, it is possible that the Pleistocene-originated ecological barriers associated with the Tanaka Line may have initiated the process of divergence between the two species without an apparent geographical barrier in this region. Under such scenario, small number of the ancestral populations of *L. decora* and *N. insignis* originally isolated of the boundary accumulated genetic differences. Following their splitting, the two species underwent past population growth as supported by joint analysis with IMa, suggesting larger effective population sizes in *N. insignis* and *L. decora* compared to the ancestor. The southwestern region of China located southeast of the Tibetan Plateau, is known to be a biodiversity hotspot due to its complex topology and unusual environmental conditions [82] and provided glacial refugia for various plant species in the Quaternary glaciations [28, 83, 84]. This region may provide sheltering for the surviving species, including the ancestor of *L. decora* and *N. insignis* [19, 23], over the periodical glaciations. Conditions during the following Quaternary glacial periods, especially the Pleistocene glacial-interglacial cycles, may have acted as stimulus to promote historically demographic shifts such as population expansions. We caution, however, that the recent spatial or demographic expansion or bottleneck for *L. decora* and *N. insignis* were not detected in the mismatch distribution estimates (Table 3, Fig. 5), which indicated their relatively stable population sizes. The different results underlie the two methods arose probably because mismatch analyses estimates the timing of sudden change in population sizes relying on parametric model of growth or decline, whereas in IMa analyses, there is longer time scale since the time of splitting and it is uncertain when the demographic changes happened in the long evolutionary history. In addition, the estimate of ancestral population sizes probably reflects post-divergence condition, resulting in biased down-estimate, for dynamics within the ancestral populations such as contractions or extinctions are not counted in IMa [85]. Further studies, particularly those employing multiple loci in coalescence times among alleles from a population to estimate population sizes through time such as extended Bayesian skyline analysis, are needed to improve examining population size change.

## Conclusions

In summary, this study provides insight into the phylogeographic patterns of two closely related species distributed along two sides of the Tanaka Line in southwest China. Although our study is based on a single chloroplast locus and one nuclear locus, we have been able to gain useful insights into the clear allopatric divergence between the two species on the population level. The extant geographic structure in the chloroplast diversity patterns observed in *N. insignis* and *L. decora* is most likely interpreted as reflecting ancestrally shared polymorphism but not post-divergence gene flow between species. In addition, our present genealogy support a major phylogeographic break in nuclear DNA between *N. insignis* and *L. decora* associated with the Tanaka Line. This phylogeographic boundary appears to represent an environmental barrier to dispersal/migration for species as previously hypothesized [31]. The establishment of differing monsoon regime during the Pleistocene on either side of the Tanaka Line probably contributed to species divergences based on our crude estimates. However, the coalescent-based methods (IMa) suffered from a lack of convergence of the lineage divergence parameter (*t*) based on the present data, thus, efforts need to be undertaken to obtain more precise estimates with multi-locus approach and perhaps parameter-rich evolutionary model implemented with Approximate Bayesian Computation to resolve their complicated evolutionary history [86].

### Availability of supporting data

The data sets of DNA sequences supporting the results of this article are available in the GenBank. GenBank accessions numbers for chloroplast DNA haplotypes are JF915764–JF915768; GenBank accessions numbers for nuclear DNA haplotypes are JF915747–JF915763.

## Additional files

Additional file 1: Table S1. Details of sample locations, sample sizes, cpDNA and *GBSSI* variation of *Leucomeris decora*. n: sample sizes, π: nucleotide diversity and Hd: haplotype diversity. Additional file 2: Table S2. Details of sample locations, sample sizes, cpDNA and *GBSSI* variation of *Nouelia insignis*. n: sample sizes, π: nucleotide diversity and Hd: haplotype diversity. Additional file 3: Fig. S1. Statistical parsimony network of genealogical relationships between 9 haplotypes derived from nDNA sequences of *Leucomeris decora* and *Nouelia insignis* without recombinations. Letters in/around circles represent haplotypes at each locus. The size of the circles corresponds to the frequency of each haplotype and each solid line represents one mutational step. Additional file 4: Fig. S2. The plots of delta K value and number of clusters (K) implemented by STRUCTURE. Additional file 5: Table S3. Maximum likelihood estimates (MLEs) of demographic parameters of the IM model between *Leucomeris decora* and *Nouelia insignis*.
