# Supplementary material for: Genetic divergence and phylogeographic history of two closely related species (Leucomeris decora and Nouelia insignis) across the 'Tanaka Line' in Southwest China
Source: BMC Evol Biol. 2015 Jul 8;15:134. doi: 10.1186/s12862-015-0374-5 (PMC4495643; doi:10.1186/s12862-015-0374-5)
Supplement: Additional file 1: Table S1. — Details of sample locations, sample sizes, cpDNA and GBSSI variation of Leucomeris decora. n: sample sizes, π: nucleotide diversity and Hd: haplotype diversity. [file 12862_2015_374_MOESM1_ESM.docx]

**Table S1** **Details of sample locations, sample sizes, cpDNA and *GBSSI* variation of *Leucomeris decora*. n: sample sizes, π: nucleotide diversity and Hd: haplotype diversity**

| Population |  | Latitude | Longitude |  | *rpl32-trnL* |  |  | *GBSSI* |  |  |
| --- | --- | --- | --- | --- | --- | --- | --- | --- | --- | --- |
| code | Location | (°N) | (°E) | n | Haplotypes | π×10^-3^ | Hd | Haplotypes (No.allels) | π×10^-3^ | Hd |
| *L.decora* |  |  |  |  |  |  |  |  |  |  |
| 1.MLA | Mengla, Yunnan | 21.990 | 101.220 | 2 | C3(2) | 0 | 0 | H8(4) | 0 | 0 |
| 2.NE | Ninger, Yunnan | 23.065 | 101.026 | 10 | C3(10) | 0 | 0 | H8(4), H10(3), H11(3), H16(8), H17(2) | 2.40 | 0.784 |
| 3.YJ | Yuanjiang, Yunnan | 23.490 | 102.011 | 10 | C3(10) | 0 | 0 | H8(3), H9(11), H10(1), H11(1), H12(3), H15(1) | 1.23 | 0.679 |
| 4.SP | Shiping, Yunnan | 23.886 | 102.267 | 10 | C2(7), C3(3) | 0.52 | 0.467 | H9(13), H10(2), H12(2), H15(3) | 0.89 | 0.563 |
| 5.ES | Eshan, Yunnan | 24.252 | 102.177 | 10 | C3(10) | 0 | 0 | H9(6), H12(8), H13(1), H15(5) | 1.32 | 0.721 |
| 6.YD-1 | Yongde-1, Yunnan | 24.021 | 99.284 | 10 | C3(10) | 0 | 0 | H9(2), H10(4), H11(11), H14(3) | 1.35 | 0.658 |
| 7.YD-2 | Yongde-2, Yunnan | 24.230 | 99.738 | 10 | C3(10) | 0 | 0 | H9(15), H11(4), H15(1) | 1.06 | 0.416 |
| 8.JD | Jingdong, Yunnan | 24.478 | 100.875 | 7 | C3(7) | 0 | 0 | H9(6), H10(1), H11(2), H12(1), H14(1), H15(3) | 1.71 | 0.791 |
| 9.BS | Baoshan, Yunnan | 24.943 | 98.868 | 10 | C2(10) | 0 | 0 | H9(10), H10(4), H11(6) | 1.33 | 0.653 |
| 10.YB | Yangbi, Yunnan | 25.476 | 99.902 | 10 | C2(10) | 0 | 0 | H9(11), H10(6), H11(2), H15(1) | 1.09 | 0.626 |
| 11.YL | Yunlong, Yunnan | 25.675 | 99.065 | 10 | C2(10) | 0 | 0 | H9(14), H10(6) | 0.61 | 0.442 |
