# Supplementary material for: Genetic divergence and phylogeographic history of two closely related species (Leucomeris decora and Nouelia insignis) across the 'Tanaka Line' in Southwest China
Source: BMC Evol Biol. 2015 Jul 8;15:134. doi: 10.1186/s12862-015-0374-5 (PMC4495643; doi:10.1186/s12862-015-0374-5)
Supplement: Additional file 2: Table S2. — Details of sample locations, sample sizes, cpDNA and GBSSI variation of Nouelia insignis. n: sample sizes, π: nucleotide diversity and Hd: haplotype diversity. [file 12862_2015_374_MOESM2_ESM.docx]

**Table S2** **Details of sample locations, sample sizes, cpDNA and *GBSSI* variation of *Nouelia insignis*. n: sample sizes, π: nucleotide diversity and Hd: haplotype diversity**

| Population |  | Latitude | Longitude |  | *rpl32-trnL* |  |  | *GBSSI* |  |  |
| --- | --- | --- | --- | --- | --- | --- | --- | --- | --- | --- |
| code | Location | (°N) | (°E) | n | Haplotypes | π×10^-3^ | Hd | Haplotypes (No.allels) | π×10^-3^ | Hd |
| *N.insignis* |  |  |  |  |  |  |  |  |  |  |
| 12.BC | Dali, Yunnan | 25.833 | 100.600 | 10 | C2(10) | 0 | 0 | H1(4), H3(16) | 0.49 | 0.337 |
| 13.DC | Dechang, Sichuan | 27.633 | 102.283 | 10 | C2(10) | 0 | 0 | H1(18), H4(2) | 0.28 | 0.189 |
| 14.DY | Dayao, Yunnan | 25.850 | 101.100 | 10 | C2(5), C5(5) | 0.62 | 0.556 | H1(10), H3(6), H4(4) | 1.13 | 0.653 |
| 15.HP | Huaping, Yunnan | 26.583 | 101.350 | 10 | C2(10) | 0 | 0 | H1(4), H3(14), H4(2) | 0.92 | 0.484 |
| 16.HTX | Zhongdian,Yunnan | 27.317 | 100.133 | 10 | C2(10) | 0 | 0 | H1(15), H3(5) | 0.57 | 0.395 |
| 17.LJ | Lijiang, Yunnan | 26.995 | 100.435 | 10 | C2(10) | 0 | 0 | H1(6), H3(14) | 0.64 | 0.442 |
| 18.LQ | Luquan,Yunnan | 25.433 | 102.483 | 10 | C2(10) | 0 | 0 | H1(20) | 0 | 0 |
| 19.MN | Mianning,Sichuan | 28.447 | 101.923 | 10 | C2(10) | 0 | 0 | H1(17), H3(1), H5(2) | 0.67 | 0.279 |
| 20.NL | Ninglang, Yunnan | 27.350 | 100.85 | 10 | C2(10) | 0 | 0 | H1(11), H3(9) | 0.76 | 0.521 |
| 21.PZH | Panzhihua,Sichuan | 26.400 | 101.767 | 10 | C2(10) | 0 | 0 | H1(15), H4 (5) | 0.57 | 0.395 |
| 22.YM | Yuanmou,Yunnan | 25.767 | 101.833 | 10 | C5(10) | 0 | 0 | H1 (18), H4(2) | 0.28 | 0.189 |
| 23.YS | Yongsheng,Yunnan | 26.567 | 100.800 | 10 | C2(10) | 0 | 0 | H1(8), H3(12) | 0.73 | 0.505 |
| 24.CJ | Chengjiang,Yunnan | 24.350 | 102.717 | 5 | C1(5) | 0 | 0 | H1(10) | 0 | 0 |
| 25.HN | Huaning, Yunnan | 24.283 | 102.850 | 10 | C4(10) | 0 | 0 | H1(20) | 0 | 0 |
| 26.ML | Mile, Yunnan | 24.683 | 103.667 | 10 | C2(7), C4(3) | 0.52 | 0.467 | H1(12), H4(2), H6(2), H7(2), H15(2) | 1.68 | 0.632 |
| 27.YANS | Yanshan, Yunnan | 23.606 | 104.341 | 7 | C2(7) | 0 | 0 | H2(14) | 0 | 0 |
