# Supplementary material for: Genetic divergence and phylogeographic history of two closely related species (Leucomeris decora and Nouelia insignis) across the 'Tanaka Line' in Southwest China
Source: BMC Evol Biol. 2015 Jul 8;15:134. doi: 10.1186/s12862-015-0374-5 (PMC4495643; doi:10.1186/s12862-015-0374-5)
Supplement: Additional file 3: Fig. S1. — Statistical parsimony network of genealogical relationships between 9 haplotypes derived from nDNA sequences of Leucomeris decora and Nouelia insignis without recombinations. Letters in/around circles represent haplotypes at each locus. The size of the circles corresponds to the frequency of each haplotype and each solid line represents one mutational step. [file 12862_2015_374_MOESM3_ESM.pdf]

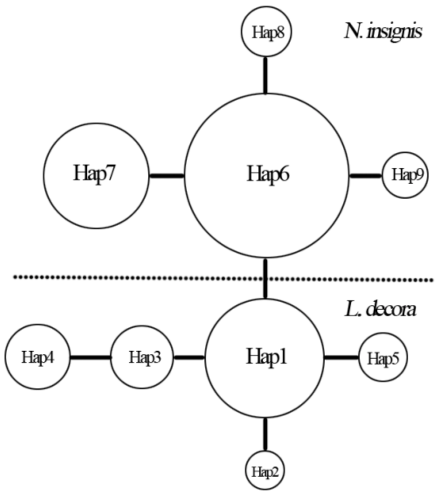

Figure S1.. Statistical parsimony network of genealogical relationships between 9 haplotypes derived from nDNA sequences of *Leucomeris decora* and *Nouelia insignis* without recombinations. Letters in/around circles represent haplotypes at each locus. The size of the circles corresponds to the frequency of each haplotype and each solid line represents one mutational step.
