# Supplementary figures and images for: Genetic divergence and phylogeographic history of two closely related species (Leucomeris decora and Nouelia insignis) across the 'Tanaka Line' in Southwest China
Source: BMC Evol Biol. 2015 Jul 8;15:134. doi: 10.1186/s12862-015-0374-5 (PMC4495643; doi:10.1186/s12862-015-0374-5)

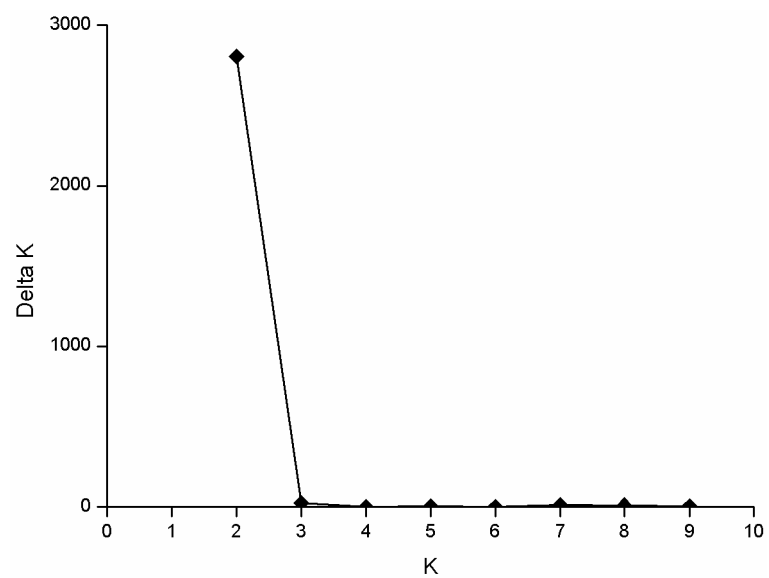

**Figure S2. The plots of delta K value and number of clusters (K) implemented by STRUCTURE.**

Supplement: Additional file 4: Fig. S2. — The plots of delta K value and number of clusters (K) implemented by STRUCTURE. [file 12862_2015_374_MOESM4_ESM.pdf]
