# Supplementary material for: Genetic divergence and phylogeographic history of two closely related species (Leucomeris decora and Nouelia insignis) across the 'Tanaka Line' in Southwest China
Source: BMC Evol Biol. 2015 Jul 8;15:134. doi: 10.1186/s12862-015-0374-5 (PMC4495643; doi:10.1186/s12862-015-0374-5)
Supplement: Additional file 5: Table S3. — Maximum likelihood estimates (MLEs) of demographic parameters of the IM model between Leucomeris decora and Nouelia insignis. [file 12862_2015_374_MOESM5_ESM.docx]

**Table S3** **Maximum likelihood estimates (MLEs) of demographic parameters of the IM model between *Leucomeris decora* and *Nouelia insignis***

| Comparison | *Θ*_1_ | *Θ*_2_ | *Θ*_A_ | *m*_1_ | *m*_2_ | *N*_1_(×10^-3^) | *N*_2_(×10^-3^) | *N*_A_(×10^-3^) | 2*N*_1_*m*_1_ | 2*N*_2_*m*_2_ |
| --- | --- | --- | --- | --- | --- | --- | --- | --- | --- | --- |
| *L*. *decora* vs *N*. *insignis* | | | | | | | | | | |
| MLE | 0.43 | 0.418 | 0.05 | 0.01 | 0.228 | 7235.68 | 7042.21 | 851.26 | 0 | 0.049 |
| Lower 95% HPD | 0.113 | 0.152 | 0.005 | 0.01 | 0.008 | 1895.98 | 2553.77 | 77.39 |  |  |
| Upper 95% HPD | 0.978 | 1.109 | 6.771 | 1.41 | 1.563 | 16444.73 | 18650.26 | 113836.2 |  |  |

*Θ*_1_, *Θ*_2_, *Θ*_A_: population sizes of *L*. *decora*, *N*. *insignis* and ancestral population, respectively; *m*_1_, *m*_2_: migration rates from *L*. *decora* to *N*. *insignis* and vice versa; *N*_1_, *N*_2_ and *N*_A_: scaled population sizes of *L*. *decora*, *N*. *insignis* and ancestral population, respectively, based on mutation rate.
